# Iontophoresis: A Potential Emergence of a Transdermal Drug Delivery System

**DOI:** 10.3797/scipharm.1108-20

**Published:** 2011-12-13

**Authors:** Vinod Dhote, Punit Bhatnagar, Pradyumna K. Mishra, Suresh C. Mahajan, Dinesh K. Mishra

**Affiliations:** 1 Mahakal Institute of Pharmaceutical Studies, Ujjain, M. P., India; 2 Division of Translational Research, ACTREC, Tata Memorial Centre, Navi Mumbai, India

**Keywords:** Drug delivery, Translational research, Transdermal therapeutic system, Iontophoresis

## Abstract

The delivery of drugs into systemic circulation via skin has generated much attention during the last decade. Transdermal therapeutic systems propound controlled release of active ingredients through the skin and into the systemic circulation in a predictive manner. Drugs administered through these systems escape first-pass metabolism and maintain a steady state scenario similar to a continuous intravenous infusion for up to several days. However, the excellent impervious nature of the skin offers the greatest challenge for successful delivery of drug molecules by utilizing the concepts of iontophoresis. The present review deals with the principles and the recent innovations in the field of iontophoretic drug delivery system together with factors affecting the system. This delivery system utilizes electric current as a driving force for permeation of ionic and non-ionic medications. The rationale behind using this technique is to reversibly alter the barrier properties of skin, which could possibly improve the penetration of drugs such as proteins, peptides and other macromolecules to increase the systemic delivery of high molecular weight compounds with controlled input kinetics and minimum inter-subject variability. Although iontophoresis seems to be an ideal candidate to overcome the limitations associated with the delivery of ionic drugs, further extrapolation of this technique is imperative for translational utility and mass human application.

## Introduction

Discovering a new medicine is a very expensive and time consuming process. However, redesigning the modules and means to transport medicines into the body are more lucrative tasks [1]. The design of a dosage form, whether in the form of a tablet, capsule, pill, cream, liquid, ointment, aerosol, injectables, suppositories or patch, to deliver the exact amount of medicine at right time to the specific target site becomes complicated if each medication were not to be delivered in an optimal and preferred manner to the individual patient [2]. Oral ingestion has long been the much convenient and commonly employed route of drug delivery. Indeed, for sustained release systems this route had received the most attention with respect to research on physiological and drug constraints as well as design and testing of products. This is because there is more flexibility in dosage form design for the oral route than there is for parenteral route. In contrast continuous intravenous infusion is recognized as a superior mode of drug administration not only to bypass hepatic “first-pass” metabolism, but also to maintain a constant and prolonged drug level in the body [3]. This mode of administration is advantageous to both direct entry of drug into the systemic circulation and control of circulating drug levels. However, such mode of drug administration entails certain risks and, therefore, necessitates hospitalization of the patients and close medical supervision of administration.

The novel drug delivery systems (NDDS) are being investigated so as to alter the body distribution of drug(s) with a view to reduce the toxicity of drug and /or deliver them more efficiently to their site of action or to improve therapeutic index. Research soon followed on ways the drug levels could be modulated and extended to derive more benefits and lower the toxicological risks from the dosage of drug [4, 5]. Novel delivery systems for non-traditional routes of administration were subsequently conceived, constructed and put to test [6]. In the last three decades a number of modern technologies including targeting concepts have emerged for successful delivery of bio-actives. The limitations have been overwhelmed by these modern technologies, which are providing effective local as well as systemic drug levels at desired sites with improved safety profiles. The increased attention on patient compliance and reduction in dose frequency has led to the development of an alternative and desirable approach of taking medicine, other than oral route for drug action, which is to deliver them through the skin [5]. Patients often forget to take their medicine, and even the most faithfully compliant get tired of swallowing pills, especially as part of daily dosage.

Skin is one of the most extensive and readily accessible organs of the human body. In modern-day pharmaceutical practice, therapeutic compounds are applied to the skin for dermatological (within the skin), local (regional) and transdermal (systemic) delivery. For transdermal delivery of drugs, stratum corneum is the main barrier layer for permeation of drug [7]. So, to circumvent the stratum corneum and to increase the flux through skin membrane, different approaches for enhancement of penetration are used. Among others things, the skin serves as a port of entry into the body for drug administration to providing continuous transdermal infusion into the systemic circulation [8]. As previously mentioned, it is one of the most extensive and readily accessible organs of the human body. As both the largest and most visible organ of the body, the skin is of unequalled importance in portraying an individual’s state of being [9]. The delivery of therapeutic agents to systemic circulation via skin requires a delivery mechanism, and many drug delivery mechanisms utilize alternative forms of energy to facilitate permeation of drugs through the skin. Thus, to provide continuous drug infusion through intact skin, transdermal drug delivery system (TDDS) has been developed for topical application onto the intact skin surface, which can deliver medicines via skin portal to systemic circulation. Delivery via the transdermal route is an interesting option in this respect since it is convenient and safe.

“The transdermal drug delivery system (TDDS) can be defined as a delivery device, which upon application on a suitable skin surface will be able to deliver the drug at controlled rate into the systemic circulation at clinically sufficient concentrations to ensure therapeutic efficacy over a prolonged period of time” [10, 11]. The benefits of using TDDS include avoidance of risks and inconveniences of parenteral therapy; improved systemic bioavailability which results in avoidance of hepatic “first-pass” effect; and TDDS avoids vagaries associated with gastrointestinal absorption due to pH, enzymatic activity, drug-food interactions, etc. Furthermore, TDDS is useful in achieving the controlled delivery of pharmaceuticals, which are relatively small in molecular size and lipophilic in nature. However, these systems are rather limited in their capability of achieving the transdermal systemic delivery of peptides, proteins and drugs which are often charged and highly hydrophilic in nature.

TDDS is associated with lacuna of low penetration rate through the outermost layer of the skin, the stratum corneum (~20 μm thick), which represents the main contributor to the skin’s impermeability. Much effort has been dedicated to improving this impermeability [12]. For this purpose, penetration enhancement is the most critical factor in transdermal systems, so as to improve flux.

**Fig. 1:**
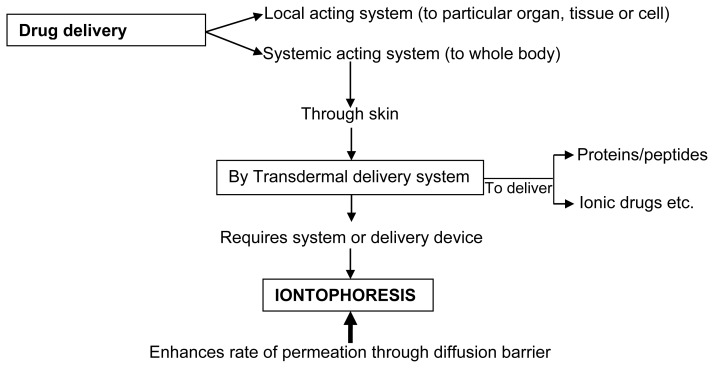
Schematic representation shows importance of Iontophoresis as penetration enhancer

Thus, percutaneous absorption is defined as penetration of substance into various layers of skin and permeation across the skin into systemic circulation [13]. One of the ways for circumventing the stratum corneum barrier includes iontophoresis as shown in Fig. 1. The percutaneous absorption is a step wise process which includes [14]:

1. Penetration: The entry of a substance into a particular layer.
2. Permeation: The penetration from one layer into another, which is different both functionally and structurally from the first layer.
3. Absorption: The uptake of a substance into systemic circulation.

### Novel Methods in Transdermal Therapeutic Systems

**Fig. 2:**
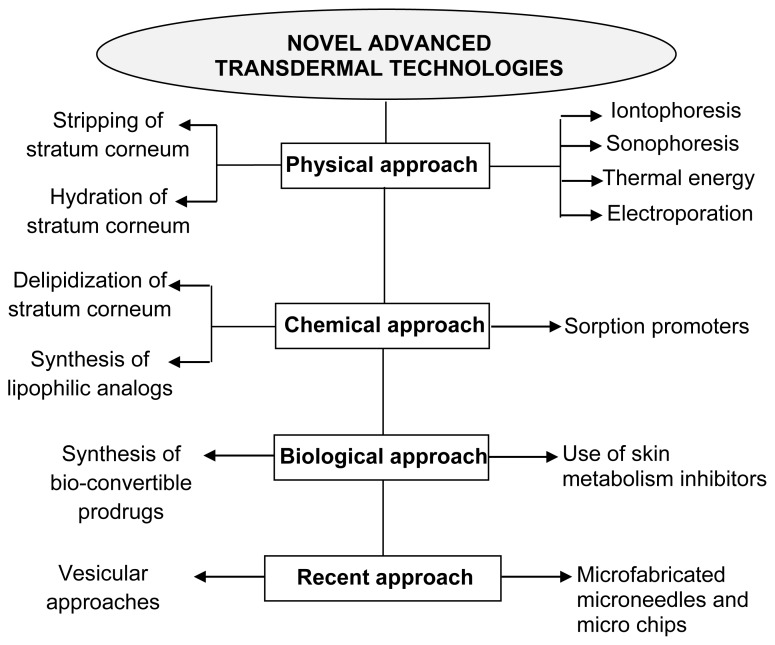
Novel advanced transdermal technologies

Newer dosage forms and drug delivery systems providing excellent improvement in drug therapy are termed novel drug delivery systems (NDDS). These are termed ‘novel’ because of recent development with satisfactory results in the field of drug delivery [15]. The primary objective of NDDS is to ensure safety and to improve efficacy of drugs as well as patient compliance. Some of these novel advanced transdermal technologies include [16] (Fig. 2):

Various technologies other than iontophoresis such as ultrasound, chemical enhancers, and electroporation have been used for enhancing transdermal drug transport. The following combinational iontophoresis techniques have been used for TDDS [17]:

- Iontophoresis + Chemicals
- Iontophoresis + Ultrasound; and
- Iontophoresis + Electoporation.

The limitations associated with chemical enhancers include unsuitability to deliver new bio-technological products like peptides, small proteins and oligonucleotides. Hence, the renaissance of interest shown toward development of a new and effective technique, iontophoresis, developed over the last decade [18].

The iontophoretic technique is suitable to improve the transdermal delivery of peptide and proteins using a lower current intensity within a short time period. Iontophoresis can be defined as “the permeation of ionized drug molecules across biological membranes under the influence of electrical current” or a technique that involves the transport of ionic or charged molecules into a tissue by the passage of direct or periodic electric current through an electrolyte solution containing the ionic molecules to be delivered using an appropriate electrode polarity [19]. Ions in solution are transferred through the skin by passing DC electrical current between two electrodes. Iontophoresis implies the use of a small amount of physiologically acceptable electric current (0.5 mA/cm^2^ or less) to drive ionic (charged) drugs into the body by using an electrode of the same polarity as the charge on the drug. The drug is driven into the skin by electrostatic repulsion. Interposition of moist pad between the electrode plate and skin is necessary for making a perfect contact, preventing any skin burns, overcoming skin resistance and protecting the skin from absorbing any caustic metallic compound formed on the metal plate surface [20]. The technique has been observed to enhance the transdermal permeation of ionic drugs by several folds and has expanded the horizon of transdermal control drug delivery for systemic medication [21]. Beside the usual benefit of TDDS, iontophoresis presents a unique opportunity to provide programmed drug delivery. This is because the permeation rate is proportional to the current density, which can be readily adjusted. Such dependence on current may also make drug absorption via iontophoresis less dependent on biological variables. While all these enhancers have been individually shown to enhance transdermal drug transport, nowadays their combinations have been hypothesized to be more effective compared to each of them alone.

### Historical background of iontophoretic process

Iontophoresis, derived from the Greek “ionto” meaning ‘ion’ and “phoresis” meaning ‘to bear,’ is a process that allows increased penetration of ionized molecule across or into the tissue by application of low electric current. Clinical application of current can be traced back to the ancient time of the golden age of the Greek civilization and was probably originated by Varatti in 1747.

The idea of applying electric current to increase the permeation of electrically charged drugs into surface tissues was probably originated by Pivati in 1747. In the eighteenth century Galvani and Vota combined the knowledge that electricity can move different metal ions and the movement of the ions produce electricity. In beginning of twentieth century Leduc introduced the term ionototherapy and formulated laws regarding this process. Furthermore, many researchers have contributed to the field of iontophoresis with great success, and some of them are represented in Table 1.

## Iontophoretic Research & Drug Delivery

Since most of the drugs showed less than adequate skin permeability in passive studies, iontophoresis was proposed to be a physical technique for enhancement of skin permeation. The release characteristics of drug from delivery system and the composition of drug delivery system can largely affect the transdermal permeation of drug molecules [39–41]. Organic solvents and surface active agents can also alter the permeability of skin and enhance the percutaneous absorption. The transdermal permeation rate increases as the drug release rate from the drug delivery system increases. Major mechanisms of enhancing drug flux through skin are:

- Iontophoresis (electrorepulsion, electromigration or Nernst plank effect)
- Electroosmotic flow
- Damage effect (current induced increase in skin permeation)

Iontophoresis enhances drug delivery across the skin by two principal mechanisms: electrorepulsion and electroosmosis. Electrorepulsion is the direct effect of the applied electric field on a charged permeant. The second mechanism, electroosmosis, results from the fact that the skin supports a net negative charge at physiological pH [42, 43].

Iontophoresis is a non invasive method used to boost high concentration of a charged substance, generally medication or bioactive agents, transdermally by repulsive electromotive force using a small electrical current applied to an iontophoretic chamber containing a similarly charged active agent and its vehicle.

For effective delivery via iontophoresis, the positively charged chamber, termed anode, will repel a positively charged chemical, while the negatively charged cathode, will repel a negatively charged chemical into the skin. In the presence of an electric field, electro-migration and electroosmosis are the dominant forces in mass transport [44].

These movements are measured in units of chemical flux, commonly μmol/cm^2^ h. This technique is based on the general principle that like charges repel each other. Thus, during iontophoresis, if delivery of a positively charged drug (D^+^) is desired, the charged drug is dissolved in the electrolyte surrounding the electrode of similar polarity, i.e. the anode in this example [45]. On application of an electromotive force the drug gets repelled and moves across the stratum corneum towards the cathode, which is placed elsewhere on the body. Communication between the electrodes along the surface of the skin has been shown to be negligible, i.e. movement of the drug ions between the electrodes occurs through the skin and not on the surface. When the cathode is placed in the donor compartment of a Franz diffusion cell to enhance the flux of an anion, it is termed cathodal iontophoresis, and for anodal iontophoresis the situation would be reversed. Iontophoresis uses a low current, and patients’ have little or no sensation during the procedure [8].

**Fig. 3:**
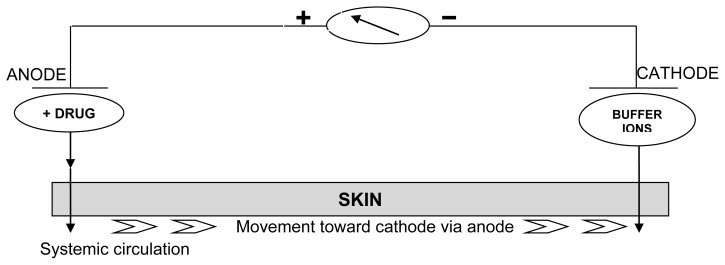
Diagram of iontophoretic technique: as current is applied the drug cations are repelled and move through the skin and eventually they are absorbed in the systemic circulation.

The basic mechanisms of ionic/molecular transport across the skin by iontophoresis is illustrated in figure 3. Like charges repel each other, hence the charged ion is repelled by a similarly charged electrode and absorbed through the skin. The skin being negatively charged at physiological pH acts as a cation selective membrane and favours movement of cations through anodal iontophoresis. Anodal iontophoresis also causes convective motion of the solvent occurring in response to movement of counter ions. This process of electroosmosis is involved in the motion of neutral compounds as well as positively charged ions. Because of the complex nature of iontophoretic delivery, a number of attempts have been made to define the rate of iontophoretic delivery.

Abramson and Gorin derived an equation to compare the iontophoresis flux to electric mobility, electroosmosis and simple diffusion. The increased flux during iontophoresis would include [29].

- Flux due to the electrochemical potential gradient across the skin;
- Change in the skin permeability due to the electric field applied; and
- Electro-osmotic water flow and the resultant solvent drag.

$${\text{J}}^{\text{ionto}}={\text{J}}^{\text{electric}}+{\text{J}}^{\text{passive}}+{\text{J}}^{\text{convective}}$$

J^electric^…The flux due to electric current application;

J^passive^…The flux due to passive delivery through the skin; and

J^convective^…The flux due to convective transport due to electro osmosis.

The Nernst-Planck equation has been used with modifications to predict iontophoretic enhancement ratios (ratio of steady state flux in presence of electric potential and in absence of potential) as the original equation lacks a term for convective electroosmotic flow [46–48] studied the contributions of osmotic flow and incorporated this fact into several equations.

### Pathways of Molecular Transport in Iontophoresis

**Fig. 4:**
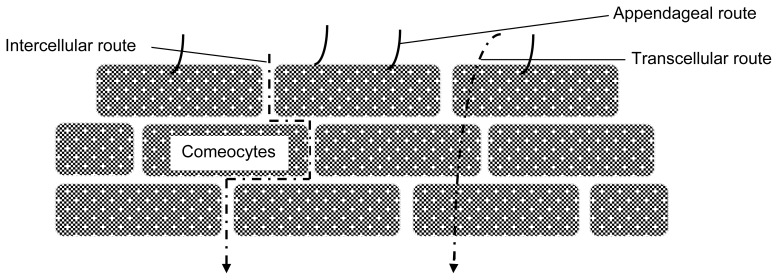
Pathways of molecular transport in iontophoresis

Percutaneous absorption may take effect simultaneously by three main pathways [49, 50] (Fig. 4.):

- Intercellular (paracellular) between the comeocytes;
- Transcellular (intracellular) through cells;
- Appendageal (shunt pathway) hair follicles, sweat ducts, secretary glands.

Ions prefer the routes of shunt pathway. Physiochemical properties of drug molecules affect the drug distribution. Hydrophilic molecules tend to localize the drug in hair follicles. On the other hand, lipophilic molecules are distributed mostly in lipid intercellular regions of the lipid membrane of epidermal keratinocytes and stratum corneum. Therefore, Transdermal iontophoresis should be termed as electrically assisted transdermal delivery.

Electroosmotic flow is a flux or bulk fluid induced by a voltage difference across a charged membrane; it is a one way flow of counter ions i.e. from anode to cathode. Therefore, cathodic delivery of anions is hindered, and thus anodic delivery of cations is assisted by it. When the delivery of large anion from the anodic compartment is more efficient than delivery from cathode, this is calledwrong-way iontophoresis. The electrorepulsion effect gives the largest enhancement to flux of small lipophilic cations [51].

### Iontophoresis as drug delivery module

The potential of this technique has been exploited for the transdermal delivery of many drugs with poor penetration properties e.g., high molecular weight electrolytes such as proteins, peptides and oligonucleotides which are normally difficult to administer except through parenteral route. It also offers a great potential for the delivery of charged peptides used as drugs. Although iontophoresis has been able to achieve significant increase in the transdermal absorption of many drugs, it has not been able to show significant permeation of larger peptides like insulin [52].

An iontophoretic drug delivery system has these basic components:

- An energy source of electronic current, which usually consists of a battery and controlled electronics;
- An active reservoir, which contains the ionic therapeutic agent; and
- An indifferent or return reservoir system, which contains an electrolyte and serves to complete the electric circuit.
- Also a control system, to monitor the overall process.

**Fig. 5:**
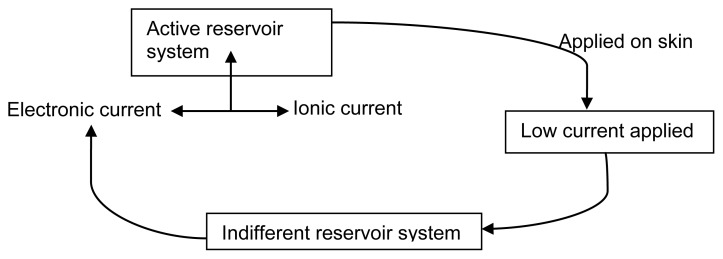
Schematic representation of iontophoretic system

When the active and indifferent reservoir systems are placed on the skin, the current source causes electronic current to flow to the active reservoir where the electronic current is transformed into ionic current. The ionic current flows through the active reservoir, through the skin, beneath the skin towards the indifferent reservoir, and back through the skin into the indifferent reservoir. At the indifferent reservoir, it is transformed back into electronic current, completing the circuit at the opposite pole of the current source [53, 54] (Fig. 5).

### Factors Affecting Iontophoresis Transport System

**Fig. 6:**
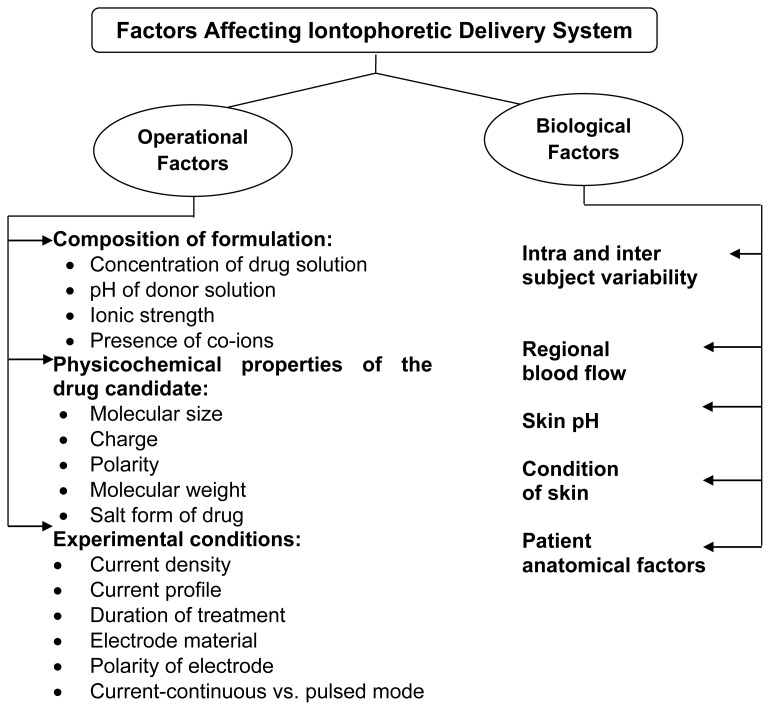
Factors Affecting Iontophoretic Delivery System

Human skin is not all the same. There are numerous differences among patient groups as well as between various regions of the body, age and ethnicity. Various factors have been shown to affect the results of iontophoresis. The following factors have to be considered (Fig. 6) [55, 56] because they may improve the delivery from the device and drug release kinetics. One of these factors, regional blood flow (dermal blood supply), determines the systemic and underlying tissue solute absorption. Blood supply, however, does not appear to affect the drug penetration fluxes through the epidermis during iontophoretic delivery. Cross and Roberts showed that solute in the upper layer of the skin following iontophoresis was comparable in anaesthetized rats and sacrificed rats. It can thus be presumed that the blood did not affect the penetration through the epidermis since the latter has no blood supply [17, 57]. Condition of skin also affects the penetrating properties of permeant. Roberts et al., studied the *in-vivo* passive diffusion of methyl salicylate using skin from different areas of the human body and observed the following rank order: abdomen > forearm > instep > heel > planter, for all subjects.

Duration and intensity of current used depends on the sensitivity of the patient. It is desirable to increase the current slowly and to remain at a predetermined level as long as the treatment required, following which the current is slowly decreased to zero [28].The time for iontophoresis ideally is 1 minute for the increasing phase and 30 seconds for the decreasing phase. The intensity of current used is between 40mA to 10mA regulated with a 2500 Ω potentiometer. Current ranging from 5 to 10mA has been found to be painless.

A major drawback of transdermal delivery systems is the potential for localized irritant and allergic cutaneous reactions. At the earlier stages of formulation development, it is, therefore, important to evaluate both drugs and excipients for their potential to cause irritation and sensitization [59, 60].

These systems are largely dependent on the ranges of parameters which affect the performance of system and such factors need to be considered (Tab. 2.) [45, 60, 61].

### Types of Iontophoretic System [62, 63]

**Fig. 7:**
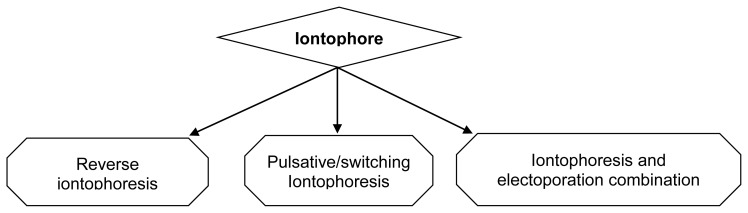
Various types of iontophoretic system

The system of drug delivery via iontophoresis can be classified in accordance to the modification and improvement done in this system which allows the uniform and predictive drug release in an effective manner (Fig. 7).

#### Reverse iontophoresis

Reverse iontophoresis, a technique in which low electric current is applied to draw intestinal fluid through the skin, is widely applied these days in devices meant for diagnostic application. This provides a convenient and non-invasive method for sampling of body fluids so as to permit simultaneous measurement of the desired substance in the body fluid and thus to monitor them efficiently. The reverse iontophoretic process applies to continuously monitor the glucose level in the blood for e.g. Glucowatch®, which is a system that provides a needleless means of monitoring blood glucose levels in diabetic patients and uses an electrical signal which is proportional to the amount of glucose in the extracellular fluid. This provides a feasible method for rapid, linear extraction of phenylalanine and for easy detection (by instruments like biosensors) of monitoring diseases like phenylketonuria. This technique not only provides non-invasive sampling but also provides filtered samples free from large molecules with ease of operation. However, this technique is useful for less tedious sampling. For it to be successful, it needs a very sensitive analytical method since the amount extracted is very low. For e.g. Caffeine, theophyline; lithium; phenytoin are successfully tried using this approach.

#### Pulsatile/switching iontophoresis

Many studies have been conducted where instead of using constant DC iontophoresis, DC in the form of short pulses have been used [63].

#### Iontophoresis and electoporation combination

Iontophoresis can also be combined with other skin penetration enhancing techniques like electoporation, which involves the application of high voltage (> 100 V) pulses for short duration (μs-ms) to increase the permeability through the skin. Electoporation is applied before iontophoresis, which causes the creation of permeablized skin because of exposure to high pulses voltage [64]. Thus, when applied after electoporation, iontophoresis helps in extending the permeablized state of the skin, resulting in the rapid onset (which is a drawback of iontophoresis alone) and sometimes increased flux [65]. The increased transport by electoporation caused by creation of electro pores as well as local field induced electrophoretic drift. Fang et al., studied the effect of electoporation on the delivery of buprenorphine and showed that on application of 300 V or 500 V pulses increased the buprenorphine flux by several folds over passive transport of it; e.g. drugs like Salmon calcitonin (SCT) and PTH combination; Tacrine hydrochloride have been successfully tried using this approach [66].

### Selection Criteria for Drug Candidate

Transdermal route of drug administration has certain inherent difficulties that make it unsuitable for a large number of drugs. The selection of suitable candidates is an important step for success of transdermal research [27, 56, 60, 67, 68].

Ideal characteristic drug should possess for the successful delivery through this approach:

- A TDDS should not cover an area more than 50 cm^2^ and the daily dose is of order of a few mg.
- The effective concentration of the drug should be low, presumably in the ng/ml.
- The half life (t_1/2_) of the drug should be short.
- The active ingredients should not cause any skin toxicity or irritation.
- As the diffusion of drug through polymer as well as skin is dependent on molecular size, the drug of low molecular size is preferred.
- The drug should have a low melting point so that it acts on normal body temperature.
- Drugs, which degrade in the GI tract or/are inactivated by hepatic first-pass effect, are suitable candidates for transdermal delivery.
- Tolerance to the drug must not develop under the near zero-order release profile of transdermal delivery.
- The candidate drug should have adequate hydrophilic and lipophilic balance to negotiate the lipid barrier of stratum corneum before being partitioned into the aqueous viable tissue.

Every system has certain benefits and shortcomings which makes it difficult to fulfill the required goal and provide the desired results in optimum conditions (Tab. 3).

Recent efforts in this technology have resulted in the design of electrodes, which avoids burns. This technique has gained acceptance for local therapy. Its application for systemic medication would require further research to elucidate an easy approach to drug delivery. The development of iontophoresis can explore and has widened the scope of transdermal delivery to the absorption of poorly absorbed ionic drugs [71–73]. There is a wide variety of drugs that has been investigated and reported recently for iontophoretic delivery (Tab. 4).

### Iontophoretic delivery of peptides and proteins

Transdermal delivery has been at the forefront of research addressing the development of non-invasive methods for the systemic administration of peptide and protein therapeutics generated by the biotechnology revolution [16, 39, 84]. Various approaches showed potential to cross the skin’s formidable barrier function. Among them iontophoresis transiently increases the skin permeability of peptides and proteins [85, 86].

Peptides and proteins play a determined role in modern therapy. Their potency and specificity make them excellent therapeutic agents; however, their physicochemical properties and stability requirements almost invariably necessitate their administration by subcutaneous, intramuscular or intravenous injection [87]. These act as effector agents that regulate and/or mediate physiological processes, serving as hormones, neuro-transmitters and signal transducing factors. Transdermal iontophoresis enables hydrophilic charged molecules to be administered through the skin in an effective, non-invasive, patient-friendly manner [88]. Controlled non-invasive administration using more patient-friendly advanced delivery technologies may combine the precision afforded by parenteral administration with improved compliance and the potential for individualized therapy [16, 39]. It has long been known that iontophoresis can administer therapeutic amounts of biologically active peptides into the body. More recent studies have shown that it is also capable of delivering structurally intact, functional proteins non-invasively into and across intact human skin [43] and becomes the most popular choice of drug delivery.

## Clinical Applications of Iontophoresis in Other Disciplines [89–91]

### Dentistry

Dentistry, probably to an even greater extent than physical therapy, has used iontophoresis with patients prior to oral surgical procedures. [22].

1. Treatment of hypersensitive dentin (e.g. in teeth sensitive to air and cold liquids) using negatively charged fluoride ions;
2. Treatment of oral ulcers ("canker sores”) and herpes orolabialis lesions ("fever blisters") using negatively charged corticosteroids and antiviral drugs, respectively; and
3. The application of local anaesthetics to produce profound topical anaesthesia, as is done in some physical therapy applications.

### Dermatology

Iontophoresis has many uses in the field of dermatology. Except for the use of lidocaine for anaesthesia and the treatment of patients with hyperhidrosis, most uses of iontophoresis in dermatology have largely been abandoned. Iontophoresis with tap water or anticholinergic compounds has been used for the treatment of patients with hyperhidrosis of the palms, feet, and axillae [91].

### Otorhinolaryngology

Iontophoresis is a preferred method for obtaining anaesthesia of the tympanic membrane prior to simple surgical procedures involving that structure. Iontophoresis of zinc has also been used for the treatment of patients with allergic rhinitis.

### Ophthalmology

Iontophoresis has been used experimentally to deliver antibiotics into the eye. The principal disadvantage of this technique is the time required for direct contact of the electrode with the eye.

### Diagnostic Applications

Iontophoretic application of the drug pilocarpine produces intense sweating, allowing sufficient amounts of sweat to be collected and analyzed. This is now accepted as the primary test in the diagnosis of cystic fibrosis.

## Conclusion

Considerations that are important for design and development of pharmaceutical products intended for application to the skin require an ideal skin penetration enhancer, for which continual research has occured over a number of decades. Although many potent enhancers have been discovered, their clinical application has been limited because of their toxic side effects. However, the recent approaches for penetration enhancement do not compromise skin barrier function as do chemical and physical penetration enhancement technique, and hence iontophoresis (as a physical enhancement technique) can serve as the better alternative. As previously defined, iontophoresis is the technique which facilitates the movement of ions across a membrane under the influence of an externally applied electrical potential difference. Iontophoresis dramatically enhances both the rate of release and the extent of penetration of the salt form of the drugs. Without iontophoresis, such charged species are largely incapable of transdermal penetration due to the skin's lipophilic nature. Iontophoresis is gaining wide popularity as it provides a non invasive and convenient means of systemic administration of drugs with poor bioavailability profile, short half life and with multiple dosing schedules. Iontophoresis, in comparison to oral route, definitely provides benefits of improved efficacy and/or reduces adverse effects.

Transdermal Technology ensures as much as 95% of a supplement reaches the cells where it is needed. Doctors around the world are calling Transdermal delivery "The delivery system of the future" and found fantastic alternative to pills and tablets. The iontophoretic delivery of macro-molecules allows the strategy for non-invasive transdermal delivery of peptide-based pharmaceuticals, and contributes to further future advancement toward recombinant DNA technology. Although iontophoresis provides many benefits and seems to be more effective than other techniques, there is a need for further research and judicious use of technology with microelectronics devices and to make it available for commercial application. Thus, iontophoresis may prove to be “a potential emergence to transdermal drug delivery”.

## Figures and Tables

**Tab. 1:** Milestones in the field of Iontophoresis

Milestones	Research
Glass et al. [22]	Conducted the most widely quoted research on iontophoresis; applied it to multiple joints on a Rhesus monkey then excised the underlying tissue to determine depth of penetration. And found that therapeutic concentrations of the drug at depths of up to 1.7 cm. The vascular network is located just below the layer of skin, therefore the drugs can be effectively delivered by this route.
Stephen et al. [23]	The first reported study on transdermal iontophoretic delivery of insulin for systemic effect was attempted to deliver regular soluble insulin to human volunteers. Iontophoresis of commercially available insulin was done on eight volunteers, but negative results were obtained even after repeating the study on three occasions. However, the investigators were able to deliver a highly ionized monomeric form of insulin to one pig and observed a decline in blood glucose levels and an increase in serum insulin levels.
Okabe et al. [24]	Applied new technique for the transdermal delivery of beta-blockers and carried out of transdermal permeation of metoprolol in human volunteers. No detectable skin damage was observed in the study.
Wearley & Chien [25]	Attempted delivery of verapamil, an anti-arrhythmic drug by using ionto-phoresis, and concluded that drugs with high systemic toxicity have a better safety potential if they are delivered through iontophoresis.
Bhatia and Singh [26]	Investigated the effect of iontophoresis on the *in-vitro* permeability of luteinizing hormone releasing hormone (LHRH) in combination with terpine through the porcine skin, which showed a significant increase in the flux of LHRH. Extensive research is now going on to develop iontophoretic systems for cardiovascular, antianxiety, and antidiabetic drugs and for inflammation and pain management.
Marro and Guy. [27]	Characterized the perm-selective properties of human and porcine skin by using mannitol as a model compound and showed that consistent isoelectric points and similar pH dependent selectivity observed for human and pigskin. Concluded that the porcine skin is an appropriate model for iontophoretic studies and found that permeation rate in iontophoresis depends on the dual parameters voltage and current density.
Figueroa et al. [28]	Demonstrated the *in-vitro* iontophoretic transdermal permeation of methotrexate across pigskin and concluded that the methotrexate transport decreases with sodium chloride content and increases with increased current density.
Anderson et al. [29]	Developed the mechanistic model to describe cathodic iontophoresis for both *in-vitro* and *in-vivo* studies by using the model drug dexamethasone/ dexamethasone phosphate. And reported that iontophoresis causes the formation of drug depot within the skin due to the exchange of drug ion with chloride ions as the ionic current carriers. Concluded that this technique will be suitable for the delivery of proteins and enzymes, which are unstable in GI tract.
Meidan et al. [30]	Demonstrated iontophoresis and permeation enhancers in combination for delivery of Buspirone. And concluded that a significant synergistic effect was found between very low-density current and permeation enhancer. Also observed that iontophoresis at 0.5 mA/cm^2^ for 24 h did not affect skin morphology, and the skin resistance reverted to its pre-iontophoretic level after discontinuation of current.
Prasad et al. [11]	Investigated iontophoresis for enhancing the transdermal transport of methotrexate by using hydrogel patches. And it was found that the transport was influenced by physicochemical properties of the system (cross-linking density of the hydrogel and copolymerisation), duration of electrical currents and the condition of the skin.
Sebastiani et al. [31]	Studied the efficacy of lactic acid as permeation enhancer for drug molecules in combination with iontophoresis, and suggested that the ionic nature of the enhancer molecules influence the overall iontophoretic flux. Lactic acid being anionic agents reduced the transport of anionic drug ibuprofen in a concentration dependent manner due to co-ionic competition; moreover, the effect of association of anodal iontophoresis showed no further enhancement.
Wang et al. [32]	Investigated the effect of iontophoresis alone and in conjunction with other approaches such as chemical enhancement, electoporation, sonophoresis and use of micro needles and ion-exchange materials. These combinations with iontophoresis may provide easier and more accurate delivery of macromolecules and poorly water-soluble compounds.
Mutalik et al.[33]	Developed a membrane-controlled iontophoretic delivery of Glibenclamide, and showed that membrane controlled system exhibited better control of hyperglycemia and more effectively reversed the diabetes mellitus complications than oral glibenclamide administration in mice.
Mutalik et al.[34]	Developed a ‘Membrane-moderated system’ for delivery of Glipizide and found that this system negligibly causes skin irritation and resulted in better control of diabetes as compared to oral administration.
Mutalik et al. [35]	Developed the matrix patch system for Glipizide delivery. Results of the *in-vivo* studies showed the patches are more effective than oral formulation on chronic application.
Kolli et al. [36]	Investigated the microneedle (MN) mediated *in vitro* transdermal iontophoretic delivery of prochlorperazine edisylate (PE) across dermatomed human skin. Application of iontophoresis in conjunction with microneedle pre-treatment resulted in an enhanced flux of drug.
Nair et al. [37]	Reported utilization of chemical penetration enhancers in conjunction with iontophoresis is regarded as the most effective method to enhance the passage of molecules across the skin barrier. A systematic approach to enhance the transdermal delivery of metoprolol tartrate and the subsequent release of the drug depot in the skin was investigated.
Takasuga et al. [38]	Investigated feasibility of transdermal delivery of tramadol, a centrally acting analgesic, by anodal iontophoresis using Ag/AgCl electrodes by *in vitro* and *in vivo* studies. The study reveals that anodal iontophoresis provides current-controlled transdermal delivery of tramadol without significant interspecies differences, and enables the delivery of therapeutic amounts of tramadol.

**Tab. 2:** Optimum levels of parameters affecting iontophoretic systems

Parameter	Optimum range	Above range	Below range
Influence of pH	pH 5.5 and below	Increasing risk for vascular reaction	The concentration of hydrogen ion increases and a vascular reaction (vasodilatation) is initiated because of C-fibre activation.
Current strength	1 mA not more than 3 min	Risk of non specific vascular reactions (vasodilatation) increases.	No resultant.
Current density	3–5 mA	Risk for unspecific electrically mediated vasodilatation	No resultant.
Molecular size and molecular weight	smaller and hydrophilic	The permeability coefficient decreases	Non specific transport.
Ionic strength & presence of other ions	—	Decrease drug delivery, as extraneous ions compete with the drug ions.	No resultant.

**Tab. 3:** Merits and demerits of transdermal iontophoretic system [60, 61, 69, 70]

Merits
It is a non-invasive technique that could serve as a substitute for chemical enhancers.
It eliminates problems like toxicity problems, adverse reaction formulation problems associated with presence of chemical enhancers in pharmaceuticals.
It may permit lower quantities of drug compared to use in TDDS, and this may lead to fewer side effects.
TDDS of many ionized drugs at therapeutic levels was precluded by their slow rate of diffusion under a concentration graduation, but iontophoresis enhanced flux of ionic drugs across skin under electrical potential gradient.
Eliminate the chance of over or under dosing by continuous delivery of drug programmed at the required therapeutic rate.
Permit a rapid termination of the modification, simply by stopping drug input from the iontophoretic delivery system.
Self-administration is possible.
Iontophoresis turned over control of local anesthesia delivery in reducing the pain of needle insertion for local anesthesia.

**Demerits**

Arrangement to protect electric shock needed.
An excessive current density usually results in pain.
Burns may be caused by electrolyte changes within the tissues.
Ionic form of drug in sufficient concentration is necessary for iontophoretic delivery.
Treatment is somewhat costly.

**Tab. 4:** List of Drugs Investigated Recently for Iontophoretic Delivery

Drug	Animal/membrane model Used	Experimental conditions	Results
Arginine & Vasopressin (AVP) [73]	Rat skin	*In vitro*: Franz diffusion cell; Ionic strength: 0.05M and 0.5M; Current: 0.5 mA/cm^2^; Time: 4h. Studied the effect of ionic strength	Enhancement ratio was found to be 6 folds at 0.5 M compared to 0.05 M ionic strength
Atenolol hydrochloride [74]	Porcine buccal mucosa	*In vitro*: Horizontal three chamber permeation cell; Current densities: 0.1, 0.2, 0.3, 0.4 mA/cm^2^; Time: 8h Studied the effect of donor concentration.	Delivery of atenolol hydrochloride increased with increase in donor concentration
Buprenorphine [75]	Human epidermal membrane	*In vitro*: Franz (vertica) diffusion cell. 0.5 mA/cm^2^; Time: 4h.	An 8 fold increase in delivery by anode than cathode
Chlorhexidine dihydrochloride [96]	Excised human skin	*In vitro*: Side by side diffusion cell; Constant iontophoresis for 1h; 0.5 mA/cm^2^	Cumulative amount of drug permeated showed a 7 times increase in drug flux by iontophoresis
Diclofenac [48]	Guinea Pigskin	*In vitro*: Current: 0.2 and 0.5 mA/cm^2^; Time: 6 h. Studied the effect of current on drug delivery.	Full plasma concentration achieved in 1 h. Drug delivery was proportional to current (371± 141 μgm / it at 0.5 mA/cm^2^ and 132 ± 62 μgm/ lt at 0.2 mA/ cm^2^).
Gentamycin [76]	White rabbits	*In vivo*: 1 mA; Time: 60 sec.	Concentration achieved in cornea and aqueous humour was 12–15 times higher than the topical eye drop.
Leuprolide (LHRH agonist) [77]	Human epidermal skin	*In vitro*: Conducted using buffers with pH: 4.5 and pH: 7.2; Current: 0.5–2.3 mA/cm^2^	Iontophoretic permeation was found to be double at pH: 7.2 than at pH: 4.5 (increased transference number was observed).
Nalbuphine (Nb) and prodrug Nalbuphine pivalate, decanoate and enduthate. [78]	Intact skin, stratum corneum stripped skin, dilipidised skin, wistar rat skin.	*In vitro*: Done to assess the effect of prodrug lipophilicity on passive and iontophoretic permeation.	Enhancement ratio highest for Nb and decreased as the lipophilicity of the prodrug increased.
Piroxicam [79]	Ventral forearm surface of human volunteer	*In vivo*: Two glass chambers; Current-0.3 mA/cm^2^ applied via phoresor II (tape stripped stratum corneum)	10 fold increased permeation.
Rotigotine [80]	Human stratum corneum	*In vitro*: Side-by-side diffusion cell; Studied the effect of drug concentration and effect of co-ions triethylamine (TEA) and tributylamine (TBA) on flux; 0.05mA/ cm^2^	Flux increased with drug concentration. With co-ions viz. TEA, flux of rotigotine increased while TBA showed no effect on flux.
Salbutamol [81]	Non rate limiting artificial membrane	*In vitro*: Release of drug from a liquid crystalline vehicle was studied.	Enhanced flux from the vehicle.
Thiocolchicoside [82]	Rabbit and human skin	*In vitro*: Glass-Franz type cell.	Enhanced flux of the drug over passive control.
Timolol maleate (TM) [83]	Excised rat, rabbit, guinea pig, mouse and human skin.	*In vitro*: Valia- Chien side by side diffusion cell. Studied the effect of species.	Iontophoretic transport highest in human skin and lowest in rabbits.
